# Kinetic and Computational Studies of CO Oxidation and PROX on Cu/CeO_2_ Nanospheres

**DOI:** 10.1007/s11244-023-01848-x

**Published:** 2023-07-31

**Authors:** Parinya Tangpakonsab, Alexander Genest, Jingxia Yang, Ali Meral, Bingjie Zou, Nevzat Yigit, Sabine Schwarz, Günther Rupprechter

**Affiliations:** 1 Institute of Materials Chemistry, TU Wien, Getreidemarkt 9/BC, 1060 Vienna, Austria; 2 College of Chemistry and Chemical Engineering, Shanghai University of Engineering Science, Longteng Rd 333, Songjiang, Shanghai People’s Republic of China; 3 University Service Center for Transmission Electron Microscopy, TU Wien, Wiedner Hauptstr. 8-10, 1040 Vienna, Austria

**Keywords:** CuO, CeO_2_, TPR, XRD, TEM, CO oxidation, PROX, Flow reactor kinetics, DFT

## Abstract

As supported CuO is well-known for low temperature activity, CuO/CeO_2_ nanosphere catalysts were synthesized and tested for CO oxidation and preferential oxidation of CO (PROX) in excess H_2_. For the first reaction, ignition was observed at 95 °C, whereas selective PROX occurred in a temperature window from 50 to 100 °C. The catalytic performance was independent of the initial oxidation state of the catalyst (CuO vs. Cu^0^), suggesting that the same active phase is formed under reaction conditions. Density functional modeling was applied to elucidate the intermediate steps of CO oxidation, as well as those of the comparably less feasible H_2_ transformation. In the simulations, various Cu and vacancy sites were probed as reactive centers enabling specific pathways.

**Supplementary Information:**

The online version contains supplementary material available at 10.1007/s11244-023-01848-x.

## Introduction

Hydrogen gas that is produced via steam-reforming of hydrocarbons or alcohols and subsequent water-gas-shift (WGS) still contains high levels of CO. For pure H_2_ streams, e.g., required for fuel cells, the CO must be removed, e.g., by preferential oxidation of carbon monoxide, CO-PROX [1–3]. PROX means that the oxidation of CO (CO + 0.5 O_2_ → CO_2_) is preferred over H_2_ oxidation (H_2_ + 0.5 O_2_ → H_2_O), avoiding the consumption of valuable hydrogen. As mentioned, this process is crucial for using the resulting H_2_ stream in polymer electrolyte membrane fuel cells (PEMFCs), because CO poisons the Pt electrodes, and significantly decreases the efficiency [3–7]. Accordingly, the CO concentration must be reduced to 10–50 ppm before feeding the H_2_ stream to the cells [8–10]. A variety of heterogeneous catalysts has shown promising performance in CO-PROX, for example, noble metals (Pd, Pt, or Rh) supported on alumina [3, 11, 12]. Nevertheless, the high-cost of precious metals inspired the search for low-cost alternatives.

Transition metal oxide catalysts containing Cu and Co have received attention, as their catalytic PROX performance was comparable to that of noble metal catalysts [3, 13–16]. Among them, CuO supported on ceria (CuO/CeO_2_) is a well-known low-cost low-temperature PROX catalyst with good activity and high selectivity for CO oxidation [3, 17–20]. Selective CO oxidation has been reported to occur in the temperature range of ~ 80–200 °C [3] and was attributed to sites at the interface of CuO and CeO_2_ [21–24]. Thus, a high dispersion of CuO clusters on ceria is clearly beneficial [25]. For PROX and low temperature CO oxidation, Co_3_O_4_ has also been frequently used [26–29].

Wang et al. prepared a catalyst of CuO particles on hollow CeO_2_ nanospheres, based on a metal-organic framework (MOF) precursor and using a template-free microwave method. In CO-PROX, 100% CO conversion was reached at about 80 °C [30]. In another work, using a hard template method [31] CuO nanoparticles were loaded on the outer surface of hollow CeO_2_ nanospheres in a layer-by-layer deposition technique. As reported in literature, the CuO surface plays a vital role as an active site for CO and H_2_ oxidation while, after the formation of CO_2_ and H_2_O, CeO_2_ provides oxygen to fill the vacancies [24, 32–35]. The oxygen transfer potential of the CeO_2_ support is also promising for other oxides [36, 37].

In the current work, CuO/CeO_2_ nanosphere catalysts were characterized by X-ray diffraction (XRD), high resolution transmission electron microscopy (HRTEM), and CO/H_2_- temperature programmed reduction (TPR), before being examined for CO-PROX in an atmospheric flow reactor. Combining experiment with density functional theory (DFT) modeling of surface structures and adsorbed CO and H_2_ on various metal and oxide sites enabled us to identify the active sites and the origin of high selectivity of CuO/CeO_2_.

## Methods

### Catalyst Synthesis and Characterization

**Fig. 1:**
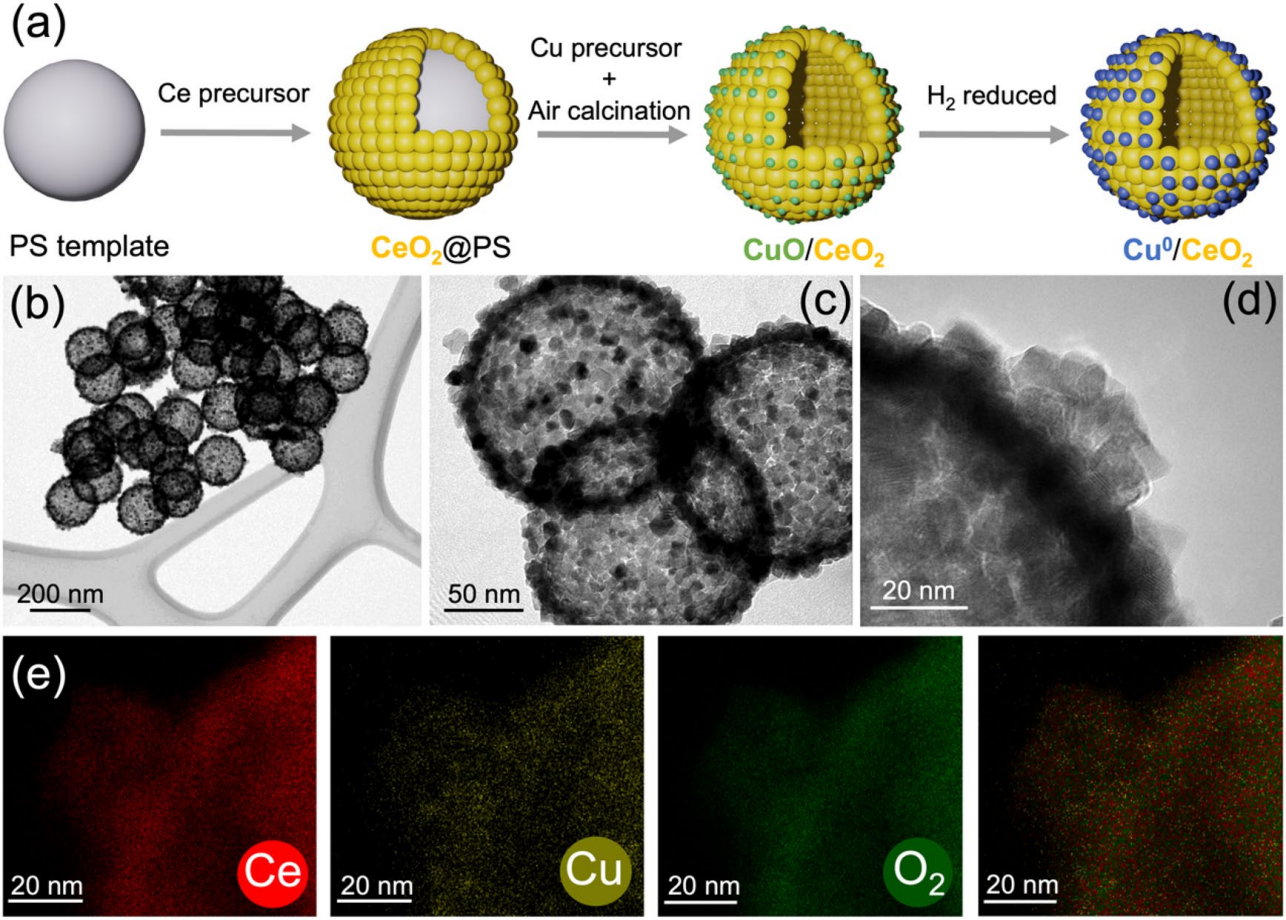
Synthesis and electron microscopy of CuO/CeO_2_: **a** schematic illustration of the synthesis, **b–d** TEM images, and **e** EDX elemental mapping of the CuO/CeO_2_ catalyst (oxidized at 300 °C)

The synthesis of CuO/CeO_2_ nanosphere catalysts was described in detail in Ref [31]. It is based on the hard template method, with CuO nanoparticles loaded on the outer surface of hollow CeO_2_ nanospheres via layer-by-layer deposition (Fig. 1a). Details of the preparation (including a final calcination at 500 °C) are presented in the Supporting Information. CO, H_2_, O_2_, and He were obtained from Messer Group GmbH, (purity 4.7 for CO and 5.0 for the others).

#### XRD

X-ray diffraction (XRD) patterns of oxidized and reduced catalysts were collected on a Philips XPERT-PRO diffractometer using Cu K-α radiation (1.5406 Å; 45 kV; 40 mA) operating in Bragg-Brentano reflection geometry with 2θ scanning from 20–90° (step size of 0.02°). Phase analyses and Rietveld refinements were performed with the HighScore Plus software (JCPDS data base) [38].

#### TEM

To study the size, morphology, and distribution of CuO/Cu^0^ particles on the ceria support, powder samples were drop-casted on carbon-coated gold grids and examined by transmission electron microscopy (TEM) in a TECNAI F20 microscope operated at 200 kV. Before TEM, the CuO/CeO_2_ catalyst was oxidized at 400 °C with 20% oxygen in He.

#### CO- and H_2_−TPR

CO- and H_2_-temperature programmed reduction (TPR) techniques were applied to characterize the reducibility of the catalysts at atmospheric pressure. Approximately 20 mg catalyst was loaded in a continuous-flow fixed-bed quartz reactor between two quartz wool plugs. Before each TPR run, contaminants were removed by pretreatment with 20% O_2_ in He (50 mL min^−1^) at 400 °C for 30 min (heating rate of 10 °C min^−1^). Samples were then cooled down to 30 °C in a flow of the same composition and purged with He for 30 min at the same temperature. The pretreated samples were then exposed at room temperature to either a mixture of 5 vol% CO in He (CO-TPR) or 5 vol% H_2_ in He (H_2_-TPR) at a flow rate of 50 mL min^−1^, before being subsequently heated to 400 °C (heating rate of 10 °C min^−1^). CO or H_2_ consumption and CO_2_ or H_2_O evolution, respectively, were analyzed by an online quadrupole mass spectrometer (QMS, Prisma Plus QMG220, Pfeiffer Vacuum) equipped with a secondary electron multiplier (SEM) detector.

### Flow Reactor Studies (CO Oxidation and PROX)

Catalytic reactions of CO oxidation and preferential CO oxidation (PROX) were carried out in the same reactor as for TPR. Concentrations of reactants and products in the outlet stream were monitored by the MS and additionally by gas chromatography (GC, Agilent 6890) using a HP-PLOT Q column, a flame-ionization detector (FID) with a methanizer and a thermal conductivity detector (TCD).

Before CO oxidation and PROX experiments, each fresh catalyst (20 mg) was pretreated as described in the following. In all cases, the heating rate was 10 °C min^−1^ and the pretreatment time was 30 min each before cool-down to room temperature. For oxidation 20 vol% O_2_ in He was used, for reduction 5 vol% H_2_ in He, with a total flow rate of 50 mL min^−1^. For both a heating rate of 2 °C min^−1^ was applied.

Temperature-dependent CO oxidation was performed for three differently pretreated catalysts: (i) oxidation at 300 °C, (ii) oxidation at 300 °C followed by reduction at 300 °C, and (iii) oxidation at 500 °C followed by reduction at 500 °C. The feed composition was 5 vol% CO and 10 vol% O_2_ in He (heating rate of 2 °C min^−1^). Additionally, to mimic PROX without H_2_, 1 vol% CO and 1 vol% O_2_ in He was supplied for a sample oxidized at 400 °C.

For PROX, two different pretreatments were applied: (i) oxidation at 400 °C, (ii) reduction at 300 °C after pretreatment). The PROX reaction was then performed in 1 vol% CO, 1 vol% O_2_, and 50 vol% H_2_ in He.

The conversion of CO (${X}_{CO}$) and O_2_ (${X}_{{O}_{2}}$), and the CO_2_ selectivity (${S}_{C{O}_{2}}$) were calculated from the GC peak areas of CO and O_2_ in the inlet and outlet of the reactor using the following formulae:

$${X}_{CO}\left(\%\right)=\frac{C{O}^{in}-C{O}^{out}}{C{O}^{in}}\times 100$$

$${X}_{{O}_{2}}\left(\%\right)=\frac{{O}_{2}^{in}-{O}_{2}^{out}}{{O}_{2}^{in}}\times 100$$

$${S}_{C{O}_{2}}\left(\%\right)=\frac{1}{2}\frac{C{O}^{in}-C{O}^{out}}{{O}_{2}^{in}-{O}_{2}^{out}}\times 100$$

To determine the activation energy (${E}_{a}$) of PROX via the Arrhenius equation, the CO conversion (in the range below 30%) was measured for the oxidized and reduced catalyst at 75, 80, 85, and 90 °C.

Furthermore, we measured the reaction orders via varying the concentration of one reactant (at 70 °C and a total flow of 50 mL min^−1^). Accordingly, to determine the CO order, the CO concentration was varied between 0.5 and 2 vol%, while the O_2_ and H_2_ concentrations were kept constant at 1 vol% and 50 vol% (yielding about 8–9% CO conversion). To determine the O_2_ order, the O_2_ concentration was varied between 0.5 and 2 vol%, while the CO and H_2_ concentrations were kept at 1 vol% and 50 vol%, respectively.

### Computational Methods

Density functional theory calculations were carried out in spin-polarized fashion using the Vienna Ab initio Simulation Package (VASP) [39] utilizing the projector augmented-wave method (PAW) [40, 41]. The generalized gradient approximation (GGA) was used in the parameterization according to Perdew, Burke, and Ernzerhof (PBE) [42]. The plane wave cutoff energy was set to 450 eV for all surface calculations. The following electron configurations were calculated explicitly, Cu(3*d*^10^ 4*s*^1^), O(2*s*^2^ 2*p*^4^), C(2*s*^2^ 2*p*^2^), and H(1*s*^1^). The strong correlations of *d* states were treated by a Hubbard model using the Dudarev formalism (DFT + U) [43]. The electronic self-consistent loop was considered converged when the energy changes became smaller than 1 × 10^−8^ eV. Atomic positions were optimized until the Hellmann-Feynman forces acting on each atom dropped below 0.02 eV/Å. According to the role of CeO_2_ described in the Introduction, our DFT study focused on the CuO surface solely.

For these calculations, the most stable CuO(111) surface was chosen, as it has the lowest surface energy (see Tables S1, S2 in the Supplementary Information (SI)). The CuO(111) surface was cut from the calculated CuO unit cell, using 8 × 8 × 8 $\varvec{k}$-points and a cut-off of 600 eV. In this study we used a 2 × 1 periodic surface supercell *p*(2 × 1)-CuO(111) with a slab thickness of 5 layers and a vacuum gap of 15 Å keeping both “bottom” layers fixed at the bulk distance. A 2 × 3 × 1 Γ-centered $\varvec{k}$-point grid was sampled for surface calculations of CuO(111). van-der Waals interactions were included using the DFT-D3 method [44].

We chose an effective $U$ value (${U}_{eff}$) of 7 eV as it tested quite reliable to describe the lattice constant and electronic properties, e.g., magnetic moment, derived from experimental work, see Fig. S1 and Table S4 of the SI.

The oxygen vacancy formation energy, ${E}_{vac}$, on a surface slab, was computed according to

$${E}_{vac}={E}_{defect/surf}+{\frac{1}{2}E}_{{O}_{2}}-{E}_{surf} \left(1\right)$$

where ${E}_{defect/surf}$ is the total energy of a surface slab with an oxygen vacancy, V_O_, and ${E}_{{O}_{2}}$ is the total energy of isolated triplet O_2_ in the gas phase. The adsorption energy, $E{\left(X\right)}_{ads}$, of an adsorbed molecule *X* (i.e., CO or H_2_) was calculated in the following way

$${E}_{ads}\left(X\right)= {E}_{X/surf}-\left({E}_{surf}+{E}_{X}\right) \left(2\right)$$

where ${E}_{X/surf}$ is the total energy of the molecule adsorbed on the surface, ${E}_{surf}$ is the total energy of the bare surface, and ${E}_{X}$ is the total energy of an isolated molecule in the gas phase. A negative ${E}_{ads}$ indicates a binding interaction. The desorption energy of molecule *X*, i.e., CO_2_ and H_2_O, ${E}_{des}\left(X\right)$ is the negative value of ${E}_{ads}\left(X\right)$. A charge analysis according to the procedure suggested by Bader [45] was carried out to evaluate the change in charge ${\Delta }q$ as described in the SI. All simulation files used in this study can be found on the ioChem-BD platform [46].

## Results

### Catalyst Characterization

Figure 1a illustrates the synthesis of the CuO/CeO_2_ nanosphere catalysts. Figure 1b–d shows TEM images, displaying CeO_2_ hollow spheres covered outside by CuO nanoparticles (partly decorated by CeO_2_). The average size/diameter of the CuO/CeO_2_ nanospheres was about 180 nm. The maps of energy dispersive X-ray analysis (EDX) (Fig. 1e and Table S1) display that cerium, copper, and oxygen are very homogeneously distributed over the hollow spheres and indicate a loading of ~ 10.5 wt% Cu or ~ 13 wt% CuO.

**Fig. 2:**
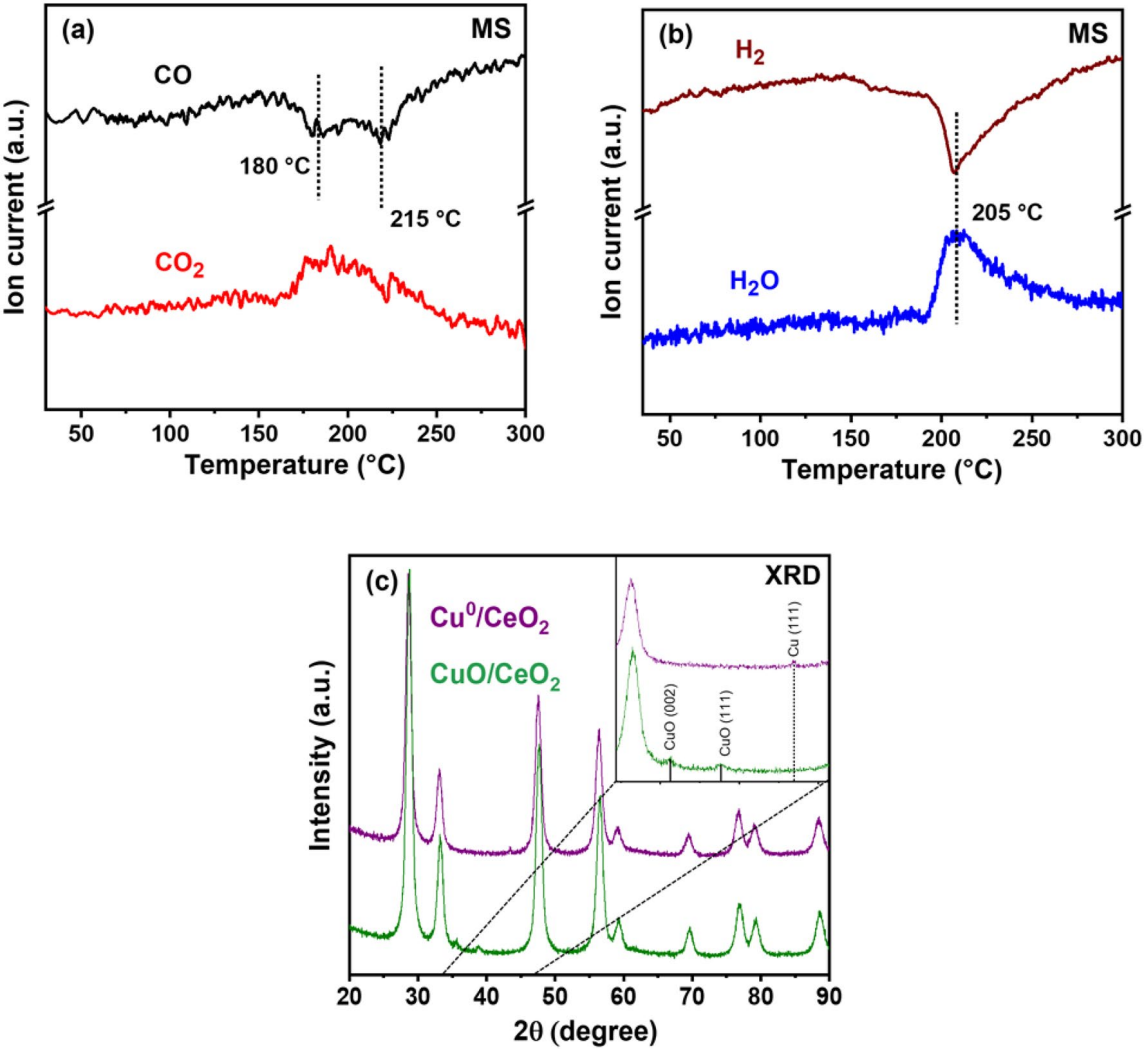
Temperature-programmed reduction and X-ray diffraction of catalysts: Mass spectrometry profiles obtained during **a** CO-TPR of CuO/CeO_2_, **b** H_2_-TPR of CuO/CeO_2_, and **c** XRD patterns of oxidized CuO/CeO_2_ and reduced Cu^0^/CeO_2_

To determine the catalyst reducibility, CO- and H_2_-TPR were performed (Fig. 2a, b). For CO-TPR, a two-step reduction (Cu^2+^ → Cu^+^ → Cu^0^) was indicated by peaks at 180 and 215 °C. In contrast, for H_2_-TPR, rather a one-step reduction (Cu^2+^ → Cu^0^) was suggested by a dominant peak at 205 °C. Overall, this behavior is quite similar to that of Co_3_O_4_ reported before [27]. As discussed below, these temperatures (representative of bulk reduction) are much higher than the typical reaction temperatures of CO oxidation and PROX. Nevertheless, surface reduction may still occur at lower temperatures.

Figure 2c shows XRD patterns of the pre-oxidized (20 vol% O_2_ in He, at 400 °C) CuO/CeO_2_ catalyst, as well as of Cu^0^/CeO_2_, obtained by the reduction at 300 °C in 5 vol% H_2_ (i.e., much higher than the 205 °C in Fig. 2b). The crystallite size was calculated using the Rietveld refinement and was 6 nm for CuO (PDF no; 00-006-2679, monoclinic) and 17 nm for Cu^0^ (PDF no; 04-009-2090, cubic). The diffractogram confirms a complete reduction of CuO at 300 °C, while CeO_2_ (PDF no; 04-003-1755, cubic) remained unaffected in both oxidized and reduced samples. The nanosphere shell is composed of CeO_2_ crystals of about 10 nm in size (9.6 and 10 nm for oxidized and reduced, respectively).

### CO Oxidation

**Fig. 3:**
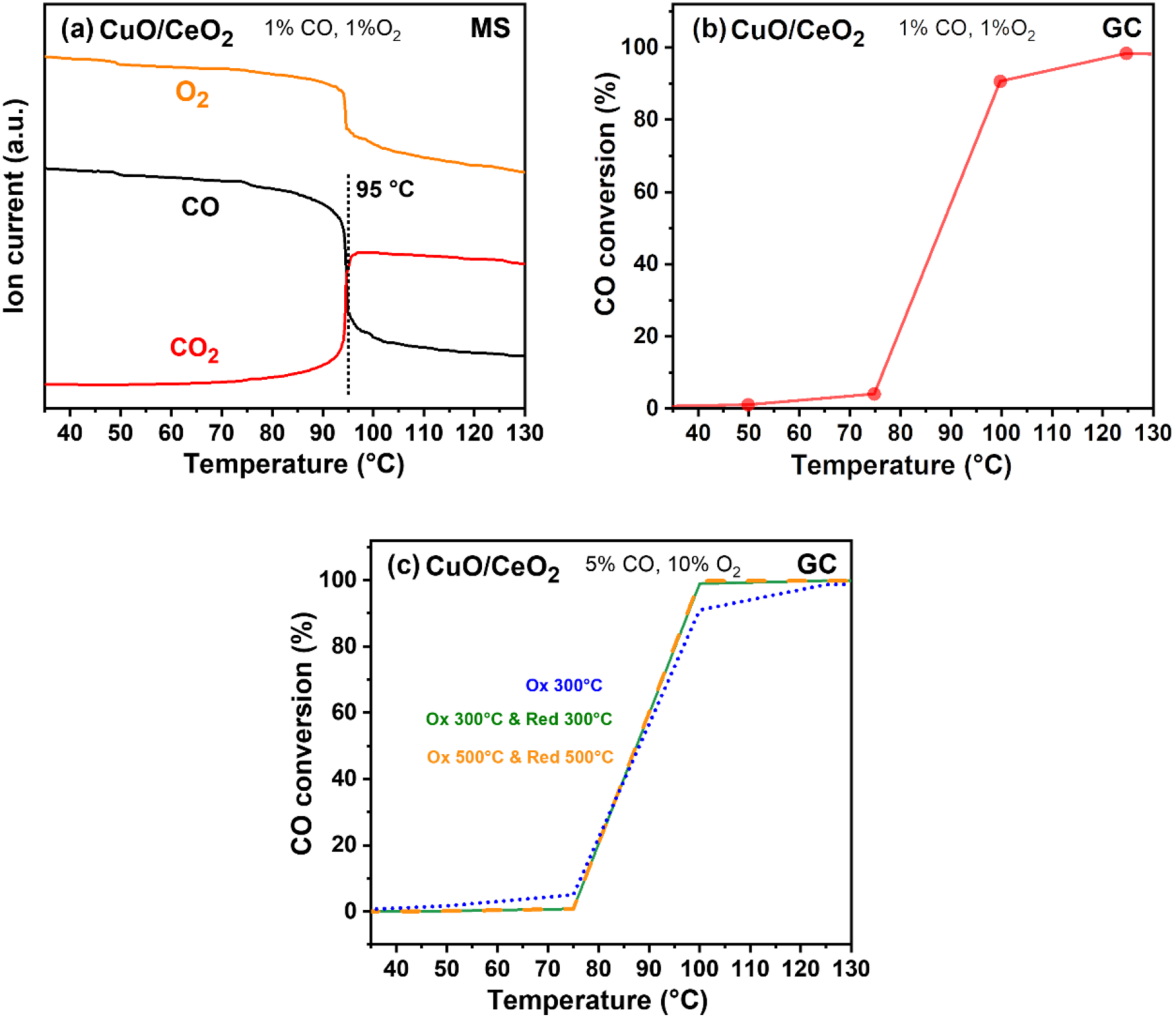
Temperature programmed CO oxidation on CuO/CeO_2_ (oxidized at 300 °C, 1 vol% CO and 1 vol% O_2_ in He) **a** MS and **b** GC of CuO/CeO_2_. **c** The same for different pretreatments: oxidized at 300 °C (blue); oxidized and reduced at 300 °C (green); oxidized and reduced at 500 °C (orange); CO oxidation (5 vol% CO and 10 vol% O_2_ in He, total flow 50 mL min^−1^)

Figure 3a, b displays results of CO oxidation (1 vol % CO and 1 vol % O_2_) on nanosphere CuO/CeO_2_, analyzed simultaneously by MS and GC. An ignition behavior [47, 48] was observed at 95 °C, leading to more than 90% CO conversion. Under similar feed conditions, a maximum CO conversion was observed at ~ 150 °C for Co_3_O_4_ [27]. For a conventional CuO/CeO_2_ morphology, a temperature of > 100 °C was needed for 90% CO conversion [49, 50]. For pure CeO_2_, CO oxidation sets in above 325 °C [51, 52]. Interestingly, for the nanosphere catalysts, the reaction onset temperature was independent of the catalyst pretreatment, i.e., the pre-oxidized and reduced (both at 300 and 500 °C) catalysts behaved identically (Fig. 3c). It seems that the same reactive phase is obtained under reaction conditions. The similar activity despite the ~ 2.5 times lower dispersion of Cu^0^/CeO_2_ may point to interface sites as active centers, as their number is less affected by particle size.

### PROX

**Fig. 4:**
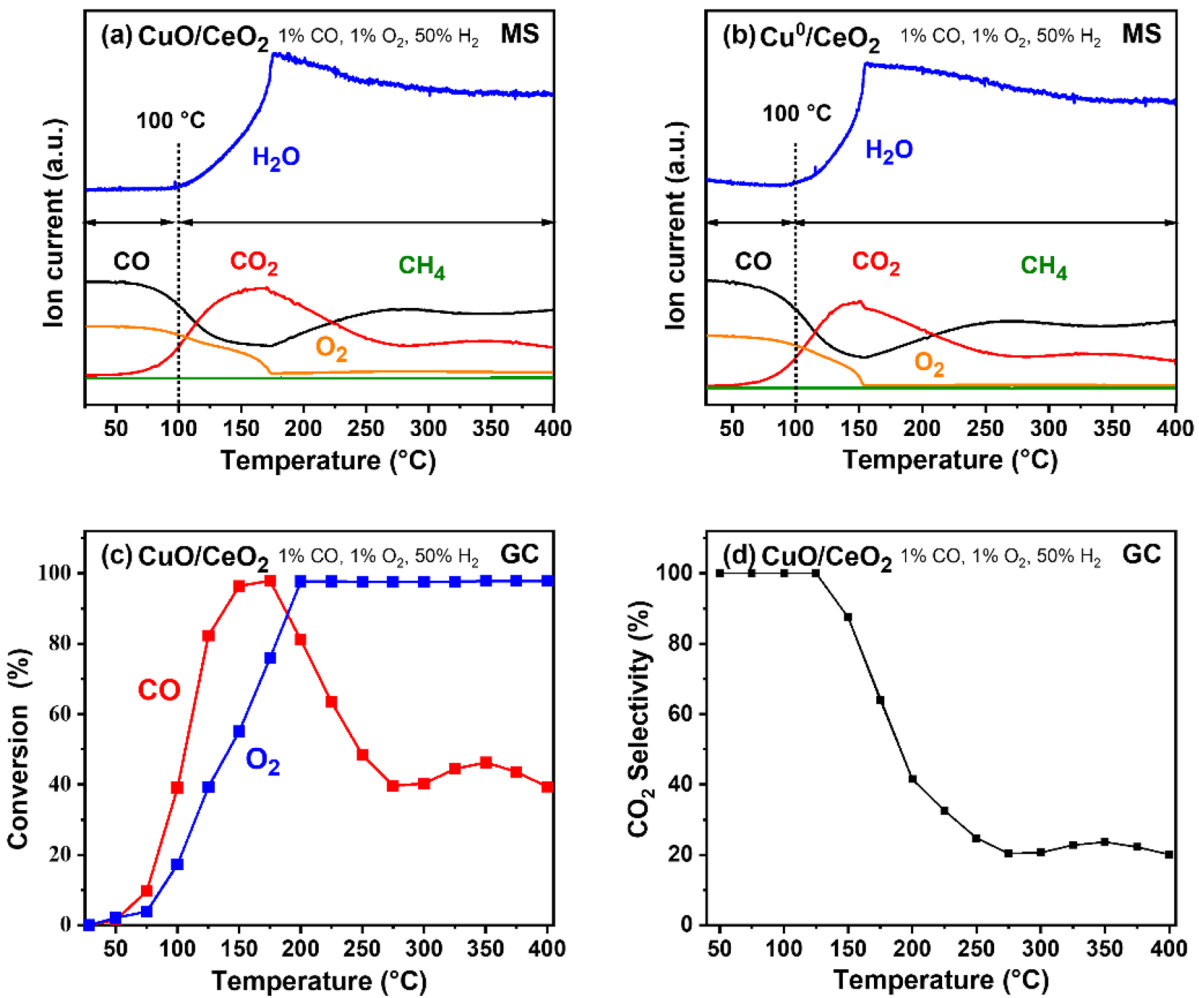
Temperature programmed PROX reaction on CuO/CeO_2_ for a reaction mixture of 1 vol% CO, 1 vol% O_2_, 50 vol% H_2_ and 48 vol% He: **a** oxidized at 300 °C and **b** after oxidation/reduction at 300 °C. PROX on oxidized CuO/CeO_2_ catalyst: **c** CO and O_2_ conversion, and **d** CO_2_ selectivity

Analogous experiments were carried out for the PROX reaction. Figure 4a, b compares MS results for CuO/CeO_2_ and Cu^0^/CeO_2_, with the reaction properties being basically identical: up to 100 °C, the catalyst shows 100% CO_2_ selectivity. Once more, the same reactive state seems to manifest under reactive conditions. GC analysis for CuO/CeO_2_ is shown in Fig. 4c, d, confirming the MS results (GC is less sensitive to small amounts of water than MS, though). Caputo et al. reported 100% CO_2_ selectivity below 144 °C on 4 wt% CuO/CeO_2_ catalyst (both oxidized and reduced) for preferential CO oxidation [53]. Above 125 °C, significant water formation sets in, leading to a drop in selectivity, although methane formation did not occur. Above 175 °C, the CO conversion ${X}_{CO}$ and CO_2_ selectivity ${S}_{C{O}_{2}}$ further decreased, likely due to the onset of a reverse Water-Gas shift reaction (CO_2_ + H_2_ ↔ CO + H_2_O), for which the Cu-based catalyst supported on CeO_2_ is known to be activated at temperatures around 200–300 °C [54, 55].

It is noteworthy that pure CeO_2_ shows PROX performance at high temperature with CO oxidation starting at around 270 °C (Fig. S2). This confirms that CuO on CeO_2_ strongly decreases the temperature of CO oxidation, enabling PROX performance at low temperature for this type of catalyst.

**Fig. 5:**
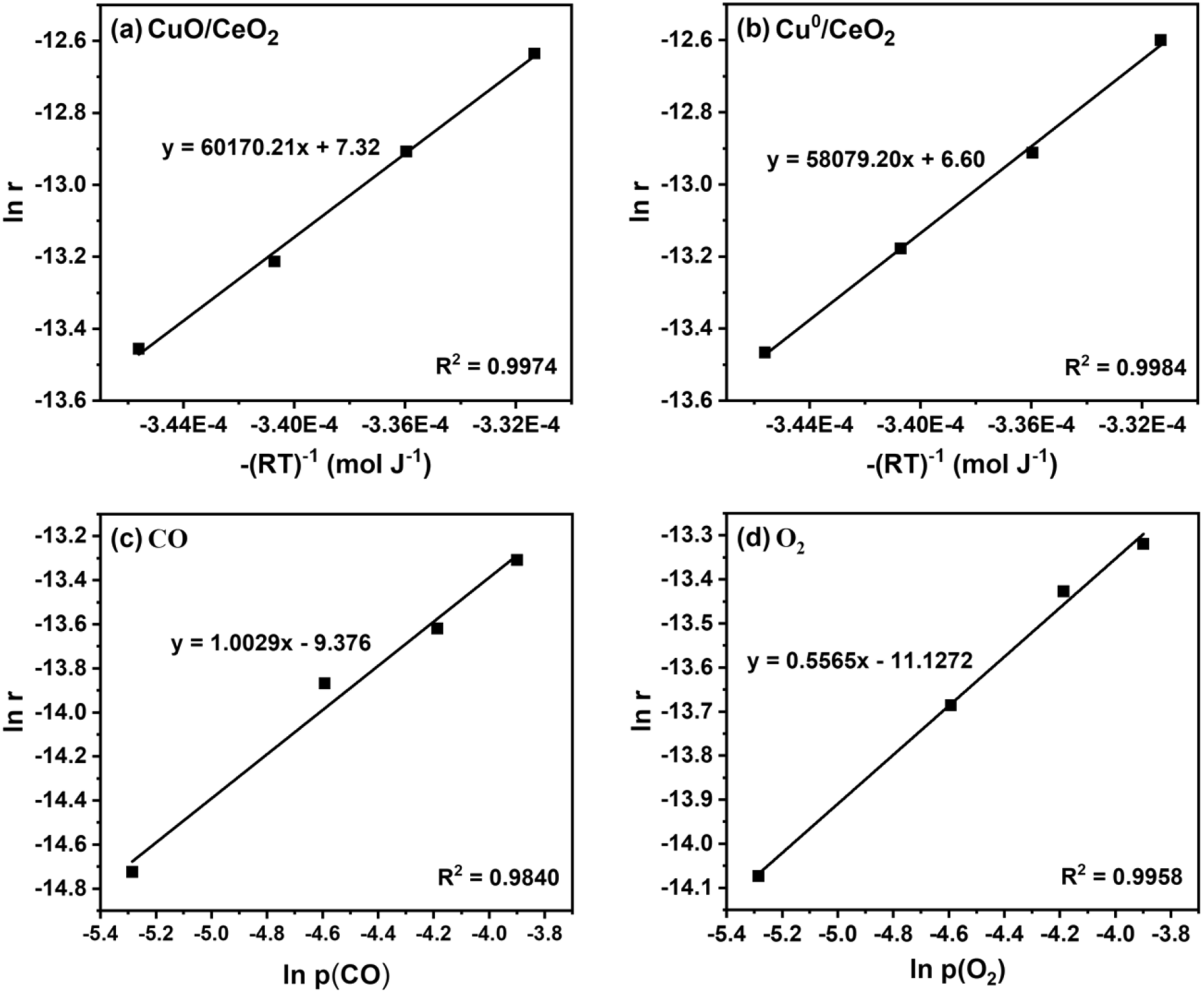
PROX on CuO/CeO_2_. Activation energies of **a** oxidized CuO/CeO_2_ and **b** reduced Cu^0^/CeO_2_ catalysts. Reaction orders of **c** CO and **d** O_2_ on CuO/CeO_2_

The Arrhenius plots in Fig. 5a, b display the activation energies (${E}_{a}$) for CuO/CeO_2_ (60.2 ± 1.7 kJ mol^−1^) and Cu^0^/CeO_2_ (58.1 ± 2.1 kJ mol^−1^); note that the fitted values for both catalysts exhibit only a small difference (2.1 kJ mol^−1^) which is within the measurement accuracy. This once more demonstrates that the initial state of the catalyst after pretreatment is not decisive for PROX. The ${E}_{a}$ values agree with those reported in the literature for selective CO oxidation over copper–ceria catalysts (${E}_{a}$ range of 55–57.2 kJ mol^−1^) [56, 57]. The ${E}_{a}$ of CO oxidation for copper-ceria catalysts is ca. 20 kJ mol^−1^ lower than that of pure CuO catalysts, confirming that ceria aids in catalytic CO oxidation [58].

For PROX, the apparent reaction order in CO and O_2_ at 70 °C (Fig. 5c, d) were determined to be ${n}_{CO}$ = 1.00 and ${n}_{{O}_{2}}$= 0.56, respectively, very close to the values expected from the reaction stoichiometry. For CO oxidation over CuO/CeO_2_ similar values of ${n}_{CO}$= 0.72–0.97 and somewhat smaller values for ${n}_{{O}_{2}}$= 0.00-0.25 were typically reported [32, 56–61].

The active state of the current CuO/CeO_2_ nanosphere catalysts has not yet been determined by in situ/operando studies [62, 63]. Nevertheless, sites at the Cu-O-Ce interface were often invoked previously to rationalize the high activity and selectivity [21–24]. In order to differentiate the contributions of Cu^2+^ and Cu^+^ on the CuO nanoparticles for low-temperature oxidation, we will take up an alternative approach herein. We employ computational modeling to rationalize the experimental results, with a focus solely on the active surface of CuO(111) [24].

### Computational Modeling

In order to obtain atomistic insights into the PROX reaction and the CO_2_ selectivity, density functional theory (DFT + U) calculations were carried out to study the formation of an oxygen vacancy, its re-oxidation by O_2_, adsorption of CO and H_2_ and reaction at various sites on a *p*(2 × 1)-CuO(111) model surface. Finally, PROX reaction pathways were evaluated, with a focus on the CO_2_ selectivity over CuO at low temperature.

#### Surface Structure and Oxygen Vacancy Formation

**Fig. 6:**
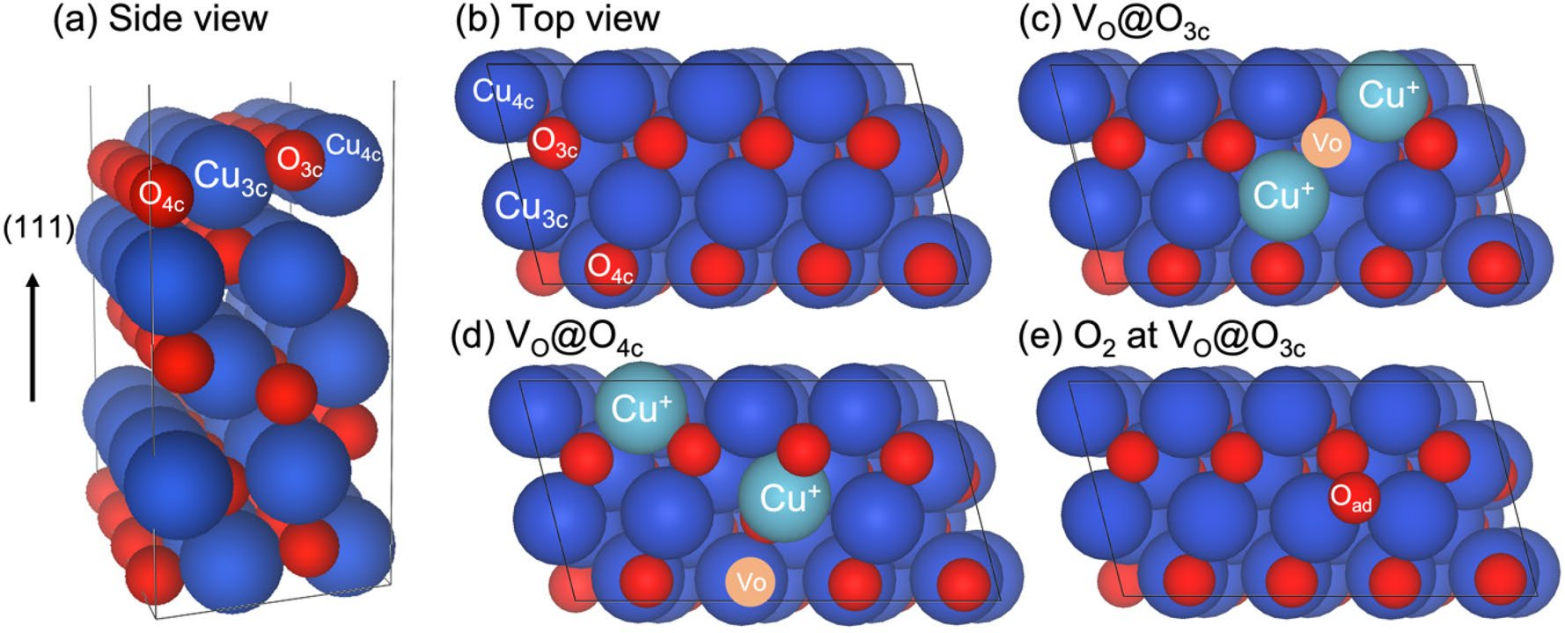
DFT study of the *p*(2 × 1)-CuO(111) structure: **a** Side view and **b** top view. Blue and red spheres represent Cu (Cu^2+^) and O atoms, while ocean green represents reduced Cu (Cu^+^). Oxygen vacancy sites V_O_ are shown in **c** at O_3c_ (V_O_@O_3c_) and **d** O_4c_ (V_O_@O_4c_). The re-oxidation of the V_O_@O_3c_ site by O_2_ is shown in **e**. The solid black line indicates the unit cell used

The CuO(111) surface model has antiferromagnetic coupled spins in which the magnetic moment per Cu remained at 0.658 ${\mu }_{B}$, alike the bulk structure. This is consistent with previous theoretical and experimental reports of 0.63 and 0.68 ${\mu }_{B}$, respectively [64, 65]. Four distinct surface sites of Cu and O atoms exist at the CuO (111) surface, as indicated in Fig. 6a, b. A three-fold coordinated O atom, O_3c_, represents the outermost surface atom while the four-fold coordinated O atom, O_4c_, represents the innermost surface atom.

CO oxidation and PROX on reducible oxides are well-known to proceed via a Mars-van-Krevelen mechanism, which involves the creation of oxygen vacancies [3, 24, 66–69]. Creating such an oxygen vacancy, V_O_, leads to two Cu atoms in the CuO(111) surface being reduced. At first, we removed the three-fold, O_3c_, and the four-fold coordinated O atoms, O_4c_, which allows a V_O_ density of $1/8$. Surfaces with V_O_ are shown in Fig. 6c, d. The oxygen vacancy formation energies ${E}_{vac}$ were calculated using Eq. (1) and are listed in Table 1. Forming an oxygen vacancy at O_3c_ site (V_O_@O_3c_) is by 0.47 eV energetically more favorable than at the site O_4c_ (V_O_@O_4c_), most likely because the latter is coordinatively more saturated by four nearest neighbors (4NN) [70, 71].

**Table 1:** Oxygen vacancy formation energies ${E}_{vac}$, of three-fold O_3c_ (V_O_@O_3c_) and four-fold O_4c_ (V_O_@O_4c_) coordinated O atoms, and adsorption energies ${E}_{ads}\left(X\right)$, of CO, H_2_, and O_2_. The desorption energies ${E}_{des}\left(X\right)$ of CO_2_ and H_2_O are included. Cu species refer to Cu_3c_. Energies are given in eV

Site	${E}_{vac}$	${E}_{ads}\left(X\right)$			${E}_{des}\left(X\right)$	
		CO	H_2_	O_2_	CO_2_	H_2_O
Cu^2+^		− 0.62	− 0.16			
Cu^+^		− 1.29	− 0.46			
Cu^0^		− 0.92	− 0.07			
O_3c_	2.41	− 1.13	− 0.87		0.28	0.77
O_4c_	2.88					
V_O_@O_3c_				− 1.99		
O_ad_		− 4.02	− 3.85		0.30	0.88

When one V_O_ is created ($\theta$=1/8), two excess electrons localize at and reduce specific Cu atoms (Cu^2+^ + *e*^*−*^ -> Cu^+^), as drawn in ocean green spheres in Fig. 6c, d. In the calculations, these reduced species are indicated by a change in the local magnetic moment and their modified atomic charge ${\Delta }q$, gaining 0.41 e^−^, Table S6. To create metallic Cu (Cu^0^) on the CuO (111) surface, a V_O_ coverage of $\theta \ge$ 5/8 is needed, resulting in a change in charge ${\Delta }\text{q}$ of 0.94 e^−^ [64].

Furthermore, we modeled the re-oxidation of the energetically favorable vacancy site (V_O_@O_3c_) by an O_2_ molecule, in addition creating the new site O_ad_ at the defective surface (Fig. 6e). Adsorption of O_2_ is strongly favorable with an energy of − 1.99 eV compared to the adsorbed CO and H_2_ species on bare surface (Sect. 3.4.2). This is in line with the calculated V_O_ formation energy. Notably, the adsorbed O_2_ molecule shows an O–O bond length of 1.50 Å, which is elongated as compared to gas phase O_2_, 1.24 Å, suggesting that adsorbed O_2_ is activated and nearly dissociated. In more detail, upon O_2_ adsorption, electrons from reduced Cu (Cu^+^) sites transfer to the oxygen moiety and occupy 2π^∗^ orbitals, leading to an elongation of the O–O bond, so that O_2_ becomes a peroxo species, O_2_^2−^ (see also the SI) [72–74]. Such peroxo species have also been discussed in the context of tuning the surface reactivity [75]. As reported in the literature, V_O_ thus plays a vital role in the adsorption of the O_2_ molecule on metal oxide surfaces [73, 76, 77].

In view of the energetically demanding V_O_ formation energies, promotion by interaction with CO or H_2_ is required. Thus, molecular adsorption of CO and H_2_, as well as surface reactions, are discussed in the next section.

#### CO and H_2_ Adsorption on the CuO(111) Surface

**Fig. 7:**
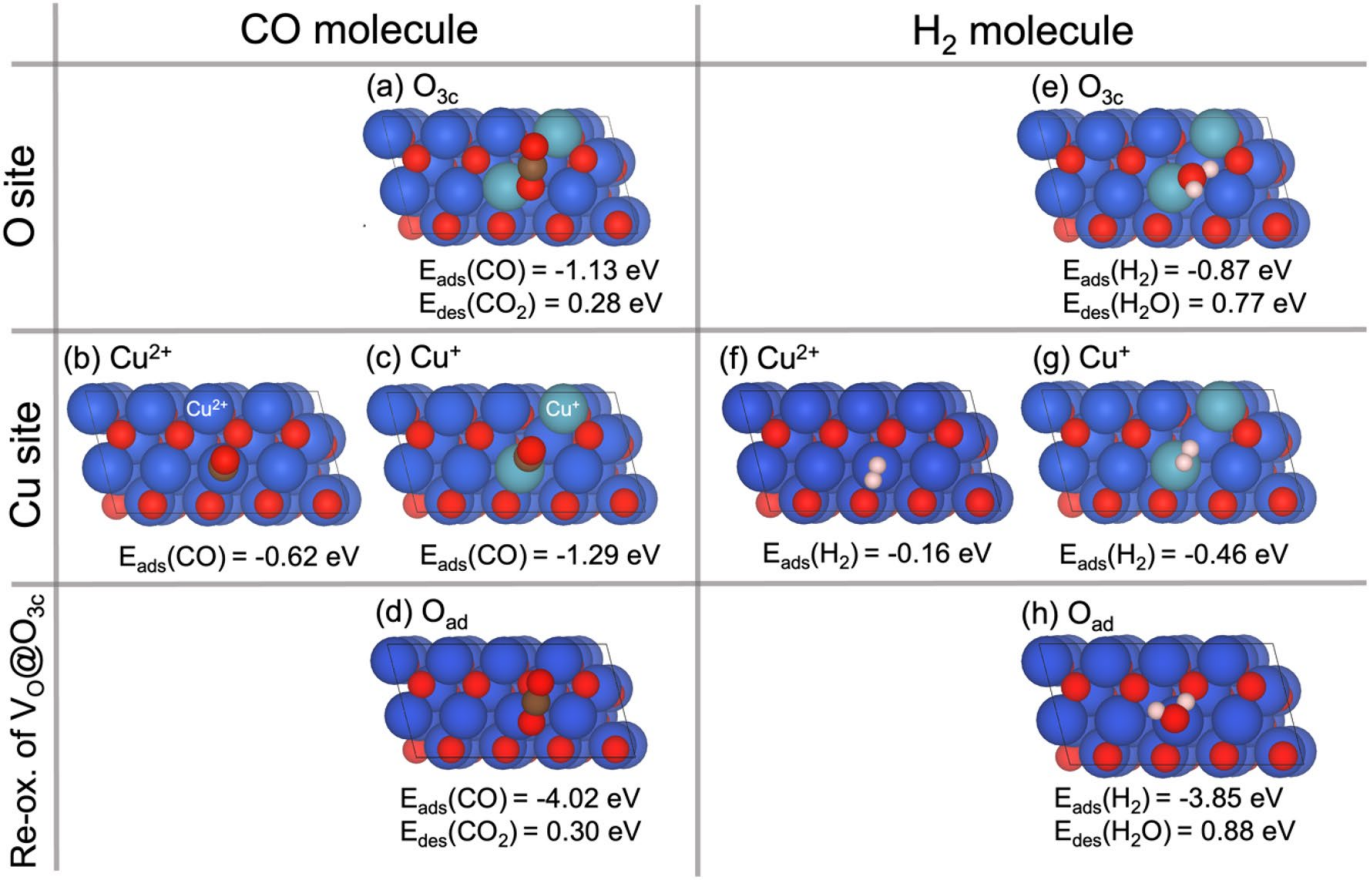
Adsorption complexes at bare surfaces and those with oxygen vacancies: Top views of CO and H_2_ adsorption on CuO_3c_ sites **a**, **e**, while **b**, **c** and **f**, **g** show adsorption on Cu sites. **d** and **h** represent CO_2_ and H_2_O formed on the extra oxygen species, O_ad_, generated from the re-oxidation of the V_O_@O_3c_ site, respectively. Blue, red, and pink spheres represent Cu (Cu^2+^), O, and H atoms, respectively, while ocean green represents reduced Cu (Cu^+^)

The stability of surface adsorption sites was probed in the presence and absence of oxygen vacancies. First, we studied the interaction of CO and H_2_ with surface oxygen species, O_3c_, and O_4c_. CO adsorbs exothermically on the activated O_3c_ site, Fig. 7a, whereas at the O_4c_ site, CO moves away from the surface. The adsorption of CO on activated O_3c_ is quite exothermic ${E}_{ads}\left(CO\right)=$ − 1.13 eV. The activated lattice O_3c_ atom is pulled out of the surface, moving by 2.43 Å and reacting with CO, forming an adsorbed CO_2_ molecule. Consequently, CO_2_ formation produces an oxygen vacancy V_O_ (V_O_@O_3c_), yielding two excess electrons which localize at Cu^2+^ to form Cu^+^ species. The adsorbed CO_2_ molecule is linear with an OCO angle of 179.2° and an average C–O distance of 1.18 Å close to the typical CO_2_ distance of 1.16 Å in the gas phase. The calculated energy ${E}_{des}$ for subsequent desorption of such a CO_2_ molecule is 0.28 eV. Keeping in mind that entropy effects are not included in the presented energetics, we can safely assume that the desorption of the CO_2_ molecule would be favorable in free energy. The adsorption of CO at Cu_2_O(100) surfaces has also been modeled, with a CO_2_ formation energy of ~ 1.7 eV [78, 79].

Alike molecular CO adsorption, the dissociative adsorption of H_2_ at O_3c_ forms a H_2_O molecule, as shown in Fig. 7e. The adsorption energy is − 0.87 eV, i.e., 0.26 eV less favorable than for CO. Furthermore, the desorption energy of the formed water molecule, ${E}_{des}\left({H}_{2}O\right)$ = 0.77 eV, is substantially larger than that of CO_2_. This rationalizes the more facile conversion of CO to CO_2_ as the formed water would remain on the surface, blocking further H_2_ activation. The calculated binding and desorption energies agree with previously published values of − 0.83 and 0.78 eV, respectively [64]. The formed water molecule binds to a surface Cu atom at a distance of 2.05 Å and the measured bond angle and average H–O distance are 108.16° and 0.99 Å, respectively. These values are close to reported theoretical work with a bond angle and H-O distance of 107.5° and 1.0 Å, respectively [64].

Adsorption of CO and H_2_ on Cu sites of the CuO(111) surface were studied next, locating two possible sites: Cu_3c_ (Cu^2+^) and reduced Cu_3c_ (Cu^+^) as listed in Table 1. The calculated adsorption energies support a spontaneous adsorption process for both molecules. CO likely adsorbs at the Cu_3c_ (Cu^2+^) site with an adsorption energy of − 0.62 eV, Fig. 7b. In contrast, CO does not adsorb at the Cu_4c_ (Cu^2+^) site and moves away, likely due to bond competition. As described above, when a vacancy is introduced (e.g., by a previous reaction with CO), two Cu_3c_ (Cu^2+^) atoms are reduced and transformed to Cu^+^. In contrast to Cu^2+^, these Cu^+^ ions adsorb CO twice as strong with ${E}_{ads}\left(CO\right)=$ − 1.29 eV, forming a CO–Cu^+^ carbonyl species, Fig. 7c. This is in line with experimental reports of Cu^+^ suggested as active centers for CO oxidation [10, 80–84] while Cu^2+^ was also reported [53]. The CO molecule binds to metallic Cu (Cu^0^) on the surface exothermically with an adsorption energy of − 0.92 eV (Fig. S4d) that is in between the other two oxidation states. However, in oxygen–containing feed (high ${\Delta }{\mu }_{O}$), Cu^0^ is hardly stable as the formation of CuO_x_ species occurs [85].

The interaction of H_2_ with Cu sites on CuO(111) was explored as well, (Fig. 7f, g and Fig. S4i of the SI), with H_2_ placed on Cu_3c_ (Cu^2+^), Cu^+^, and Cu^0^ atoms. The H_2_ molecule binds exothermically to the Cu^+^ site with an adsorption energy of − 0.46 eV, 0.30 eV stronger than the Cu^2+^ site, and agrees with a previously reported value of ${E}_{ads}$ = − 0.47 eV [86]. In contrast, it shows very weak adsorption at Cu^0^, ${E}_{ads}\left({H}_{2}\right)=$ − 0.07 eV, which is in good agreement with a previous study of H_2_ adsorption on metallic Cu(111)$, {E}_{ads}\left({H}_{2}\right)$ = − 0.07 eV [87]. Overall Cu^+^ adsorbs both H_2_ and CO better than the other oxidation states of Cu.

During the reaction the formed vacancies on the surface will be spontaneously replenished by gaseous O_2_ forming an active oxygen species at the surface, O_ad_. Thus, the adsorption of CO and H_2_ at O_ad_ was studied accordingly. O_ad_ reacts with CO and spontaneously forms CO_2_ with a strongly exothermic reaction energy of − 4.02 eV (Fig. 7d). In the same way, the adsorption of H_2_ also forms H_2_O with an adsorption energy of − 3.85 eV (Fig. 7h). As expected, the O_ad_ species at the V_O_–CuO (111) surface is quite reactive in the oxidation process. A study of oxygen vacancy rich La_0.8_Sr_0.2_CoO_3_ also suggested that the presence of nearby V_O_ enhances the CO oxidation/interaction with adsorbed O_ad_ species [73]. Note that the possibility of hydroxyl formation [88] from H_2_ adsorption at O_3c_ was also observed at a V_O_–CuO(111) surface (see Fig. S5). To complete the catalytic cycle, the formed species CO_2_ and H_2_O desorb from the surface with energies of 0.30 and 0.88 eV. This again indicates a strongly favored CO_2_ desorption, which rationalizes the experimentally observed high CO_2_ selectivity. A possible reaction mechanism is described in the following section.

#### Suggested Reaction Pathways on the CuO(111) Surface

**Fig. 8:**
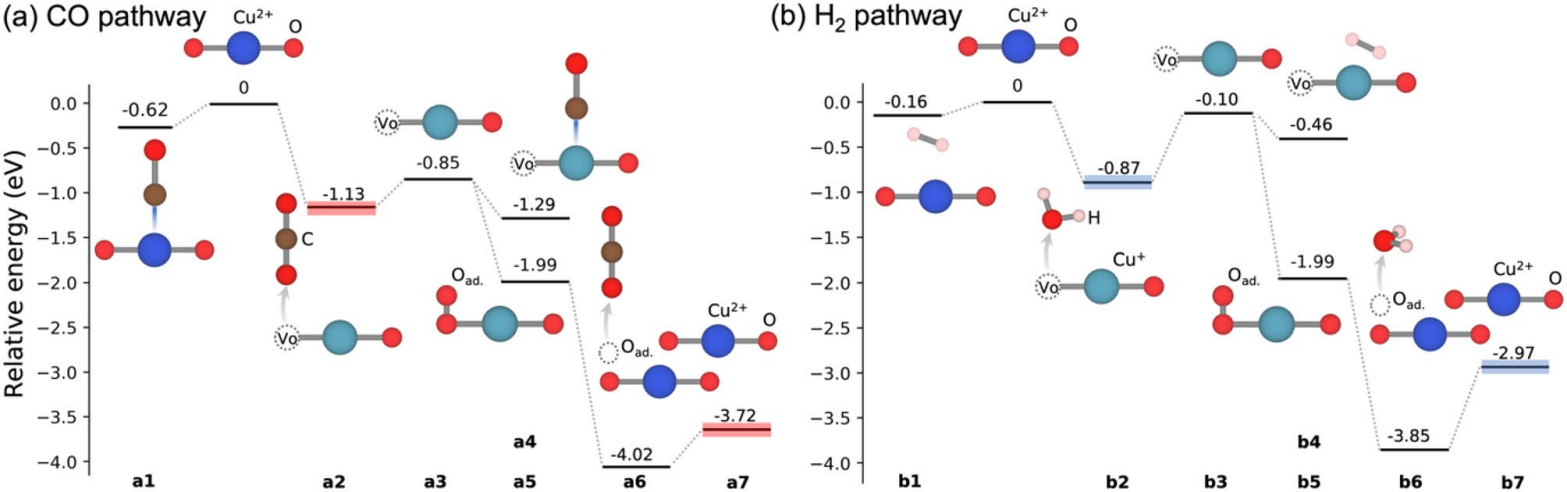
Reaction pathways of **a** CO and **b** H_2_ oxidation. Energy baseline of CO_2_ and H_2_O products are marked in red and blue. Energies are reported relative to the bare surface and CO/H_2_ in the gas phase. Blue, red, and pink spheres represent Cu (Cu^2+^), O, and H atoms, respectively, while ocean green represents reduced Cu (Cu^+^)

Following the results on the interaction of O_2_, CO, and H_2_ at CuO(111) we suggest two reaction pathways (Fig. 8) based on a Mars-van-Krevelen (MvK) mechanism [24, 89, 90]. Adsorbing CO and H_2_ molecules from the gas phase on Cu^2+^ (**a1, b1**) is slightly exothermic, but to form CO_2_/H_2_O and continue with the catalytic cycle the adsorption at an oxygen site is needed. Adsorption at O_3c_ forms CO_2_ (**a2**)/H_2_O (**b2**) and one V_O_, with the CO_2_ formation being slightly preferred. The formed CO_2_ easily desorbs into the gas phase, while H_2_O desorption is substantially more challenging. These energies are compatible with the experimental results where CO_2_ is preferentially produced up to ~ 100 °C, whereas above H_2_O formation sets in and dominates > 175 °C (Fig. 4). Note that the transformation of CO to CO_2_ and H_2_ to H_2_O on activated O_3c_ occurs without barrier as a downhill process except for the very first activation. In line with previous studies [91, 92], our nudged elastic band calculations [93, 94] showed these barriers to be smaller than ~ 0.5 eV for CO and smaller than ~ 0.7 eV for H_2_, around/below the “barrier” for the desorption process. The reaction rate will thus be controlled by the desorption process, for which we have determined a significant difference between H_2_O and CO_2_.

The formed vacancy V_O_ has two Cu^+^ neighbors allowing further adsorption of CO (**a4**)/H_2_ (**b4**). Alternatively, O_2_ may repopulate the vacancy site (**a5-b5**), resulting in an added oxygen species, O_ad_. At this special oxygen another CO or H_2_ may adsorb and form CO_2_ (**a6**) or H_2_O (**b6**). Yet again, CO_2_ (**a7**) desorbs more favorably by 0.6 eV into the gas phase than H_2_O (**b7**), completing the reaction cycle.

## Summary

CuO/CeO_2_ nanosphere catalyst particles are active in CO oxidation and CO-PROX and were tested in the present work via experimental and computational approaches. CO oxidation ignited at 95 °C, while under PROX conditions, 100% selectivity to CO_2_ was maintained up to 100 °C, overall showing that the nanosphere catalyst works fairly well. Oxidative or reductive pretreatments, initially forming Cu^2+^ or Cu^0^ moieties, respectively, yielded no discernable difference in catalytic steady-state performance and activation energies. Hence, the same active catalyst phase is formed under reaction conditions.

Using DFT modeling, the energetics of likely reaction pathways on Cu oxide surfaces was further studied, indicating a conventional Mars-van-Krevelen (MvK) type mechanism. Upon CO adsorption and conversion to CO_2_, oxygen vacancies on Cu oxide surfaces were easily formed, rather independent of the oxidation state of neighboring Cu centers. For further adsorption, we determined that Cu^+^ is the preferred binding site, with CO adsorption being much stronger than H_2_ adsorption. Molecular O_2_ adsorption and subsequent activation proceeds at a vacancy, creating highly active O species. Upon interaction with CO the formed CO_2_ binds only weakly at the surface allowing for rapid desorption and freeing sites for further turnover, promoting CO_2_ selectivity as observed experimentally. The adsorption/activation of H_2_ is less feasible and the resulting water binds much stronger to the surface. This explains the undesired water formation only at high CO conversions and above 100 °C. Ceria is certainly beneficial due to its oxygen vacancies, but the support was not considered herein. Operando studies are planned for the future to elucidate active phases under reaction conditions.

## Supplementary Information

Below is the link to the electronic supplementary material.

Supplementary material 1 (PDF 1255 kb)

## Declarations

### Data Availability

DFT data supporting the research is available in the ioChem-DB database.

10.19061/iochem-bd-6-230.
