# Supplementary material for: Kinetic and Computational Studies of CO Oxidation and PROX on Cu/CeO2 Nanospheres
Source: Top Catal. 2023 Jul 31;66(15-16):1129–42. doi: 10.1007/s11244-023-01848-x (PMC10505120; doi:10.1007/s11244-023-01848-x)
Supplement: Supplementary file 1 — Supplementary material 1 (PDF 1255 kb) [file 11244_2023_1848_MOESM1_ESM.pdf]

Supplementary Information

Kinetic and Computational Studies of CO Oxidation and PROX on Cu/CeO_2 Nanospheres

Parinya Tangpakonsab[1], Alexander Genest[1], Jingxia Yang[2], Ali Meral[1], Bingjie Zou[2], Nevzat Yigit[1], Sabine Schwarz[3], Günther Rupprechter[1]*

[1]Institute of Materials Chemistry, TU Wien, Getreidemarkt 9/BC, 1060 Vienna, Austria

[2]College of Chemistry and Chemical Engineering, Shanghai University of Engineering Science, Longteng Rd 333, Songjiang, Shanghai, P.R. China

[3]University Service Center for Transmission Electron Microscopy, TU Wien, Wiedner Hauptstr. 8-10, 1040 Vienna, Austria

* Correspondence: guenther.rupprechter@tuwien.ac.at

Keywords

CuO, CeO_2, TPR, XRD, TEM, CO oxidation, PROX, flow reactor kinetics, DFT

Catalyst synthesis and characterization

The synthesis of the CuO/CeO_2 nanosphere catalysts is based on the hard template method, with CuO nanoparticles loaded on the outer surface of hollow CeO_2 nanospheres by layer-by-layer deposition, as shown in Fig. 1a.

Chemicals. All analytical grade chemicals were used without further purification. Sodium borohydride ($NaBH_4$), cerium nitrate hexahydrate ($Ce(NO_3)_3{\cdot}6H_2O$), 37.0% formaldehyde solution (CH_2O), and phenylethylene(C_8H_6) were supplied by Shanghai Macklin Biochemical Co. Ltd. Hexamethyl tetramine ($C_6H_{12}N_4$) was obtained from Sinopharm Chemical Reagent Co. Ltd. Copper nitrate trihydrate ($Cu(NO_3)_2{\cdot}3H_2O$) and ethanol (C_2H_6O) were provided by Aladdin Chemistry Co, Ltd.

Synthesis of CeO_2@PS. The polystyrene (PS) template particles with a diameter of about 200 nm were synthesized by the unsaponifiable emulsion polymerization method according to the literature [1]. Then, PS powders (0.12 g) were ultrasonically dispersed in 60 mL of anhydrous ethanol, forming a PS suspension. $Ce(NO_3)_3{\cdot}6H_2O$ (0.608 g) and hexamethyl tetramine (0.98 g) were put into a beaker with 300 mL deionized water and fully dissolved by stirring and further mixed with the above-mentioned PS suspension. Next, the mixture was transferred to a 500 mL round bottom flask and reacted for 6 h at 80 °C. After cooling the solution to room temperature, the sample was centrifuged and washed with distilled water and EtOH and dried overnight at 60 °C, obtaining CeO_2@PS.

Synthesis of CuO/CeO_2. CeO_2@PS (0.361 g) was ultrasonically dispersed in 80 mL of aqueous-ethanol mixed solution (1:1 in volume ratio) and $Cu(NO_3)_2{\cdot}3H_2O$ (0.0564 g) was added and fully dissolved. Under constant stirring, aqueous sodium borohydride solution (0.1 g $NaBH_4$ in 40 mL H_2O) was added dropwise. After 30 min stirring, the products were centrifuged and washed with distilled water and ethanol and dried at 60 °C for 12 h. The samples were further calcined to remove the PS template particles, as follows: First, the heating rate was 5 °C min^{-1} and the temperature was ramped up to 310 °C from room temperature and kept at 310 °C for 2 h. Then, the temperature was ramped up to 330, 350, and 370 °C at 0.5 °C min^{-1}. At the same time, each temperature at 330, 350, and 370 °C was maintained for 2 h, and finally, the heating rate was 5 °C min^{-1} from 370 to 500 °C and kept at 500 °C for 2 h.

Table S1 EDX analysis of CuO/CeO_2 nanosphere catalysts.

Element	Mass %	Atomic %
O K	21.7	67.8
Ce L	65.5	23.3
Cu K	10.5	8.3
Other	2.3	0.6

Surface energies

A surface energy (γ) is the energy needed to create a surface and indicates the surface stability: the lower the surface energy the more stable is the surface. Based on theory, the low-index (111) surfaces of Cu_2O and CuO structures were reported to have the lowest surface energy (γ) as shown in Tables S2, S3, respectively. It can be concluded that the (111) surface is the most stable surface of Cu_2O and CuO [2–7].

Table S2 Surface energies (γ) of low-index Cu_2O surfaces.

Surfaces	γ (J/m^2)		
	PBE+U[a]	PBE[b]	PBE[c]
(111)	1.08	0.76	0.76
(110): Cu	1.24	-	-
(110): Cu-O	1.54	-	1.04
(100): Cu	1.62	-	-
(100): O	-	-	1.26

[a]reference [2] [b]reference [5] [c]reference [4]

Table S3 Surface energies (γ) of low-index CuO surfaces.

Surfaces	γ (J/m^2)		
	PBE+U[a]	PBE[b]	PBE+U[c]
(111)	0.75	0.74	0.72
(011)	0.94	0.93	-
(110)	1.00	1.29	-

[a]reference [3] [b]reference [6] [c]reference [7]

Geometry optimization and magnetic properties

Stoichiometric bulk CuO and Cu_2O were optimized with PBE+U where the choice of U_{eff} was adjusted from 0 to 9 eV. To determine the U_{eff} parameter, we calculated a lattice constant of bulk Cu_2O and CuO as a function of U_{eff} and have found that U_{eff} = 7 eV yields a lattice constant in agreement with the experimental value (see Table S4). For the case of CuO, with U_{eff} = 7 eV, we obtained antiferromagnetic spin coupling where the averaged local magnetic moment of 0.658 μ_B is in good agreement with previous theoretical and experimental studies. On the other hand, a nonmagnetic moment is predicted for Cu_2O.

It is worth noting that U_{eff} = 7 eV can reproduce both electronic structures, *i.e.*, lattice constant and magnetic moment, for both stoichiometric CuO and Cu_2O. This is also reported in the theoretical work of Mishra et al.[2, 3]. The optimized structures and their parameters, and magnetic properties of CuO and Cu_2O, are shown in Figure S1a-b and Table S4. CuO and Cu_2O possess monoclinic and cubic cell structures, respectively, in which averaged Cu-O distances are 1.96 Å for CuO and 1.85 Å for Cu_2O. This is also confirmed by the match of X-ray diffraction (XRD) patterns of CuO (solid blue) and Cu_2O (solid orange) calculated in this study with experimental XRD patterns (dashed lines), as depicted in Fig. S1c [8, 9]. The calculated XRD peak of CuO(111) is in the same range, between 35-40° of 2θ, as our experimental CuO/CeO_2 result, Figure 2c.

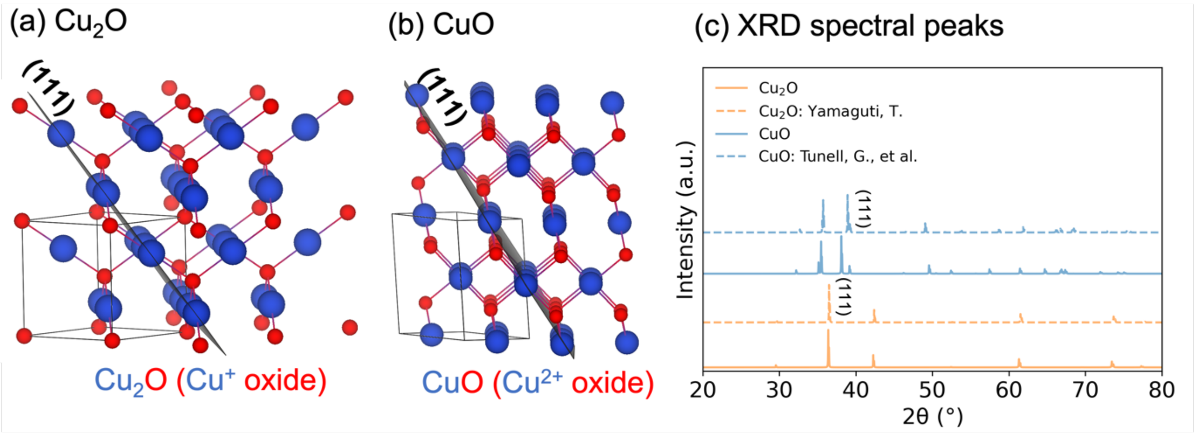

Fig. S1 Optimized structures of modeled (a) Cu_2O and (b) CuO. Blue and red spheres represent copper and oxygen atoms, respectively. Their corresponding XRD peaks are shown in (c). XRD peaks from experimental observations are plotted as dashed lines for ease of comparison [8, 9].

Table S4 Optimized parameters with PBE+U where U_{eff} = 7 eV of bulk CuO and Cu_2O. Experimental data of Cu_2O [10] and CuO [11, 12] are included for ease of comparison.

Bulk	$\boldsymbol{a}$ (Å)	$\boldsymbol{b}$ (Å)	$\boldsymbol{c}$ (Å)	$d_{Cu\text{-}O}$ (Å)	$\lvert m_s \rvert$ (μ_B)
Cu_2O calc.		$\boldsymbol{a} = \boldsymbol{b} = \boldsymbol{c} = 4.28$		1.85	0
Cu_2O exp.		$\boldsymbol{a} = \boldsymbol{b} = \boldsymbol{c} = 4.27$		-	-
CuO calc.	4.62	3.51	5.14	1.95	0.658
CuO exp.	4.6837	3.4226	5.126	1.95	0.69$\pm$0.05

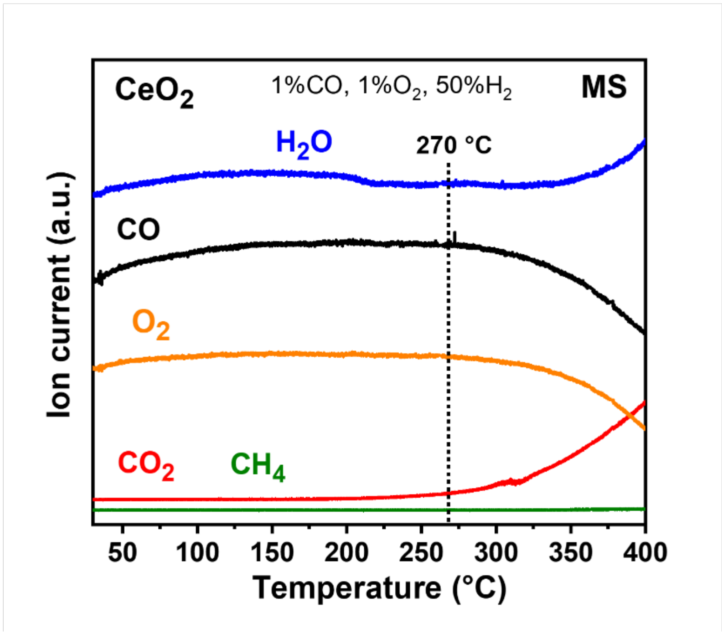

Fig. S2 PROX reaction for a feed of 1 vol % CO, 1 vol % O_2, 50 vol % H_2 and 48 vol % He (total flow 50 mL min^{-1}) on pure CeO_2 support.

Bader charge analysis

Bader charge analysis [13] was performed to quantify the change of charge Δq when a defect is introduced (*i.e.,* oxygen vacancy V_O). The change of charge is computed by

$$\Delta q = q_{surf/defect} - q_{bare\ surf} \, ,$$

where $q_{surf/defect}$ and $q_{bare\ surf}$ are the calculated Bader charges of the bare surface and defective surface, respectively.

To indicate the oxidation state of the Cu atom, the Bader charges in CuO, Cu_2O, and Cu bulk structures were computed *via* $\Delta q = q_{bulk} - 11$, where q_{bulk} is the Bader charge of Cu atoms in bulk structure and 11 indicates the number of Cu valence electron as shown in Fig. S3 and Table S5. The calculated Bader charge change after V_O formation is presented in Table S6.

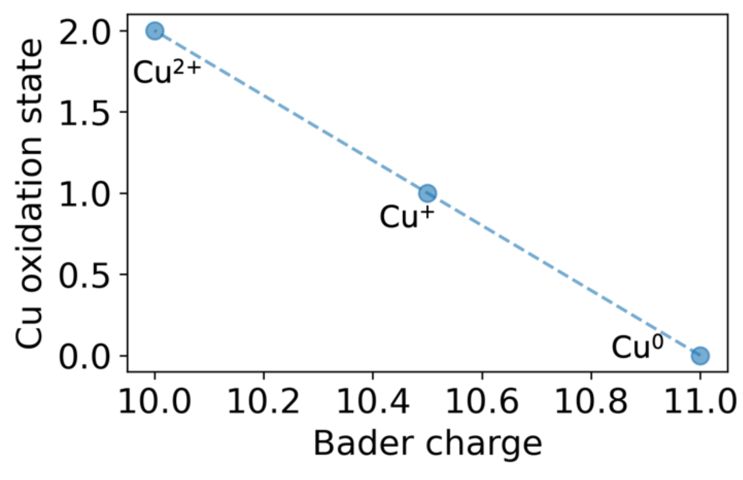

Fig. S3 Cu oxidation states as a function of Bader charge analysis calculated from stoichiometric CuO, Cu_2O, and Cu bulk, respectively.

Table S5 The Bader charge q_{bulk} of Cu, Cu_2O, and CuO bulk structures: Bader charge changes per Cu atom (Δq) and corresponding oxidation states of Cu.

System	Bader charge q_{bulk} (e^-)	Bader charge change Δq (e^-)	Oxidation state
Cu	11.00	0.00	Cu^0
Cu_2O	10.51(10.46)[a]	0.49	Cu^+
CuO	9.97(9.90)[a]	1.03	Cu^{2+}

[a]reference [7]

Table S6 Average Bader charge changes (Δq) of reduced Cu sites (Cu^+) after introduction of three-fold O_{3C} and four-fold O_{4C} oxygen vacancies at the *p*(2x1)-CuO(111) surface.

Method	U_{eff} (eV)	Average Δq of reduced Cu sites (e^-)	
		V_O@O_{3c}	V_O@O_{4c}
PBE+U[a]	7	0.41	0.41
PBE+U[b]	7	0.44	0.44

[a]this study [b]reference[7]

Molecular CO and H_2 adsorption

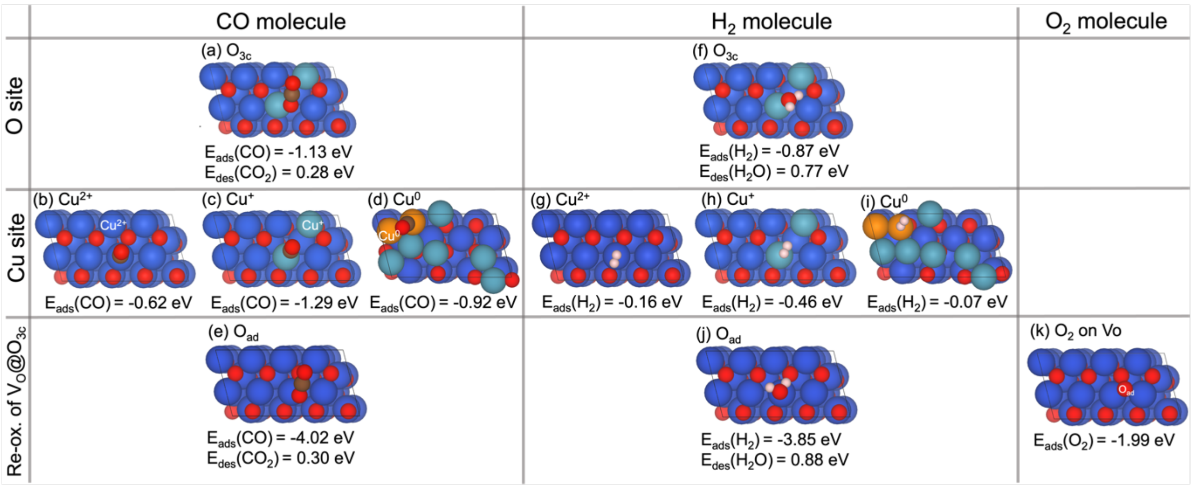

Fig. S4 Top views of CO and H_2 adsorption on O_{3c} site (a,f) while (b-d) and (g-i) show adsorption on the Cu site. k) shows the re-oxidation of the V_O@O_{3c} site by gaseous O_2 forming the extra oxygen species, O_{ad}. e) and j) represent formed CO_2 and H_2O on O_{ad}. Blue, red, and pink spheres represent Cu (Cu^+), O, and H atoms, respectively. Reduced Cu atoms Cu^+: (Cu^{2+} -> Cu^+) and Cu^0: (Cu^+ -> Cu^0) are drawn as ocean green and orange spheres, respectively.

Re-oxidation of a V_O@O_{3c} site by gaseous O_2

The re-oxidation of a vacancy site (V_O) was calculated by adsorbing a stable O_2 molecule (triplet oxygen) to the V_O@O_{3c} site of the reduced *p*(2x1)-CuO(111) surface. Table S7 shows adsorption energy $E_{ads}(O_2)$, Bader charge q, and oxygen bond distance d_{O-O} of gas phase and adsorbed O_2 species, respectively.

Table S7 The adsorption energy, Bader charge q, and O-O bond distance d_{O-O} of gas phase and adsorbed O_2 molecule on the V_O@O_{3c} site at *p*(2x1)-CuO(111) surface.

	$E_{ads}(O_2)$ (eV)	q (e^-)	d_{O-O} (Å)
gas phase O_2	-	6.00	1.24
adsorbed O_2	-1.99	6.55	1.50

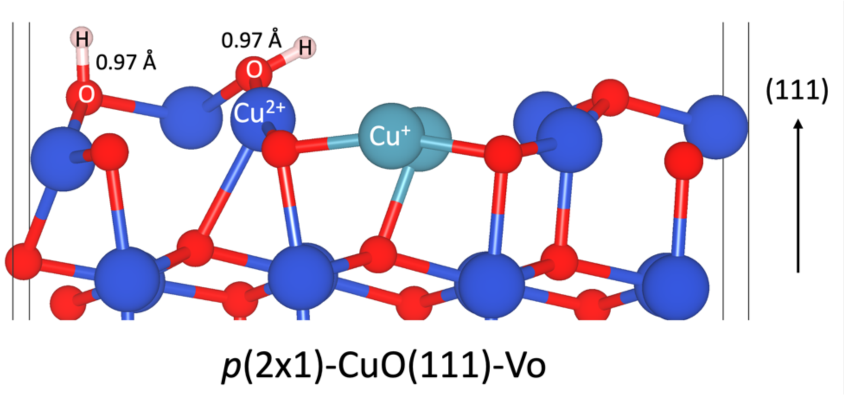

Fig. S5 Formation of OH at the CuO(111) surface with one oxygen vacancy V_O.

References

1. Cao S, Zou B, Yang J, et al (2022) Hollow CuO-CeO_2 Nanospheres for an Effectively Catalytic Annulation/A^3-Coupling Reaction Sequence. ACS Appl Nano Mater 5:11689–11698. https://doi.org/10.1021/acsanm.2c02666
2. Mishra AK, Roldan A, de Leeuw NH (2016) A density functional theory study of the adsorption behaviour of CO_2 on Cu_2O surfaces. J Chem Phys 145:44709. https://doi.org/10.1063/1.4958804
3. Kumar Mishra A, Roldan A, De Leeuw NH (2015) CuO Surfaces and CO_2 Activation: A Dispersion-Corrected DFT+*U* Study. The Journal of Physical Chemistry C 120:2198–2214. https://doi.org/10.1021/acs.jpcc.5b10431
4. Ferrer MM, Fabris GSL, de Faria B v, et al (2019) Quantitative evaluation of the surface stability and morphological changes of Cu_2O particles. Heliyon 5:e02500. https://doi.org/10.1016/j.heliyon.2019.e02500
5. Islam MM, Diawara B, Maurice V, Marcus P (2009) Bulk and surface properties of Cu_2O: A first-principles investigation. Journal of Molecular Structure: THEOCHEM 903:41–48. https://doi.org/10.1016/j.theochem.2009.02.037
6. Hu J, Li D, Lu JG, Wu R (2010) Effects on Electronic Properties of Molecule Adsorption on CuO Surfaces and Nanowires. The Journal of Physical Chemistry C 114:17120–17126. https://doi.org/10.1021/jp1039089
7. Maimaiti Y, Nolan M, Elliott SD (2014) Reduction mechanisms of the CuO(111) surface through surface oxygen vacancy formation and hydrogen adsorption. Physical Chemistry Chemical Physics 16:3036–3046. https://doi.org/10.1039/C3CP53991A
8. Yamaguti T (1938) An Investigation on Oxidation of Crystal Surfaces with Electron Diffraction Method, II. Copper Single Crystals. Nippon Sugaku-Buturigakkwai Kizi Dai 3 Ki 20:230–241. https://doi.org/10.11429/ppmsj1919.20.0_230
9. Tunell G, Posnjak E, Ksanda CJ (1935) Geometrical and Optical Properties, and Crystal Structure of Tenorite. Z Kristallogr Cryst Mater 90:120–142. https://doi.org/doi:10.1524/zkri.1935.90.1.120
10. Werner A, Hochheimer HD (1982) High-pressure x-ray study of Cu_2O and Ag_2O. Phys Rev B 25:5929–5934. https://doi.org/10.1103/PhysRevB.25.5929
11. Åsbrink S, Norrby L-J, IUCr (1970) A refinement of the crystal structure of copper(II) oxide with a discussion of some exceptional e.s.d.'s. urn:issn:0567-7408 26:8–15. https://doi.org/10.1107/S0567740870001838
12. Yang BX, Thurston TR, Tranquada JM, Shirane G (1989) Magnetic neutron scattering study of single-crystal cupric oxide. Phys Rev B 39:4343. https://doi.org/10.1103/PhysRevB.39.4343
13. Tang W, Sanville E, Henkelman G (2009) A grid-based Bader analysis algorithm without lattice bias. Journal of Physics Condensed Matter 21:. https://doi.org/10.1088/0953-8984/21/8/084204
